# Case report: Metastatic *BRAF V600E*–mutated adult Wilms’ tumor with robust response to *BRAF/MEK* inhibitor therapy

**DOI:** 10.3389/fonc.2024.1376270

**Published:** 2024-07-15

**Authors:** Matthew R. Kroll, Cherry Au, Jessica Slostad, Trevor N. Christ, Sam G. Papas, Alan Tan

**Affiliations:** ^1^ Department of Internal Medicine, Rush University Medical Center, Chicago, IL, United States; ^2^ Division of Hematology, Oncology, and Cellular Therapies, Rush University Medical Center, Chicago, IL, United States; ^3^ Department of Pharmacy, Rush University Medical Center, Chicago, IL, United States; ^4^ Division of Surgical Oncology, Rush University Medical Center, Chicago, IL, United States

**Keywords:** precision oncology, *BRAF/MEK* inhibition, Wilms, rare tumors, *BRAF V600E*

## Abstract

Nephroblastoma or Wilms’ tumor (WT) is the most common pediatric renal malignancy but rare in adults. Treatment protocols for adults are typically extrapolated from pediatric guidelines, but there are no standard guidelines for adults due to the rarity of the disease. However, next-generation sequencing has led to new therapeutic options for adult WT patients. We present the first case to our knowledge of a recurrent adult WT treated with dual *BRAF/MEK*–targeted therapy, which showed initial robust clinical response and was well tolerated.

## Introduction

Nephroblastoma or Wilms’ tumor (WT) is the most common pediatric renal malignancy. Adult WT is a rare entity, with an incidence of 0.2 per million, and treatment protocols for adults are typically extrapolated from pediatric guidelines (1). These guidelines recommend a multidisciplinary approach to WT, involving surgery, chemotherapy, and radiation. Overall, survival of adult WT treated with pediatric treatment protocols is 82%; however, metastatic recurrent WT has poor prognosis due to lack of standard guidelines (2, 3). Without a standard of care treatments for adults, this tumor is challenging to diagnose and treat, and the clinical outcomes remain inferior compared to the pediatric population. However, next-generation sequencing (NGS) has led to new therapeutic options for adult WT patients. Multiple reports have characterized mutations in the *BRAF* proto-oncogene in cases of WT (3–5). There are two cases reported in the literature of successful *BRAF*-targeted therapy with more than 1 year of successful response to treatment: a 6-year-old boy with *BRAF/MEK* inhibitor dabrafenib and trametinib and 51-year-old male with vemurafenib monotherapy (3, 4). We report the first case to our knowledge of metastatic *BRAF V600E–*mutated adult WT treated using combination *BRAF/MEK* inhibitor therapy.

## Case report

A 34-year-old female presented with abdominal pain and was subsequently diagnosed with right renal mass consistent with stage II nephroblastoma (WT). She underwent right radical nephrectomy and seven cycles of adjuvant chemotherapy with dactinomycin and vincristine. End of treatment positron emission tomography (PET) scan showed no evidence of disease. Thirteen months later, the patient presented with abdominal pain and distention, nausea, and vomiting. Computed tomography abdomen and pelvis imaging revealed an 11-cm mass in the upper abdomen and a 14-cm mass in pelvis invading the small bowel mesentery. Biopsy of the mass confirmed recurrent WT. The patient then presented to our institution for further management.

**Figure 1:**
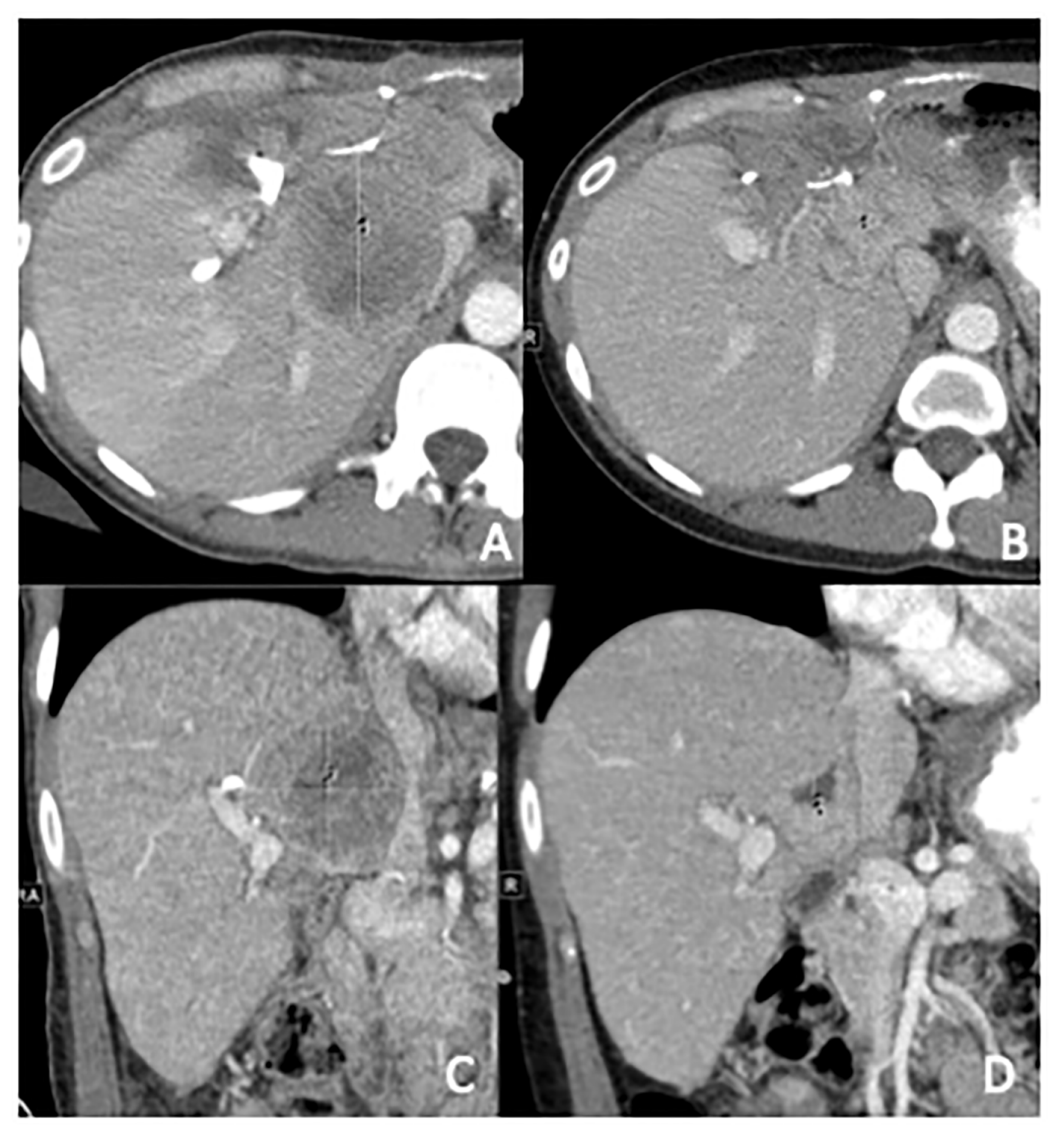
Computed tomography abdomen with contrast, in axial and coronal planes prior to initiation **(A, C)** and 3 months post-initiation **(B, D)** of dabrafenib and trametinib. In **(A, C)**, the metastases measure 5.1 cm × 5.6 cm × 5.4 cm and compress the IVC. In **(B, D)**, the metastases shrunk to 2.7 cm × 2.7 cm × 2.6 cm and the Inferior Vena Cava (IVC) is no longer compressed.

She underwent a resection of the large pelvic mass, en bloc resection of the liver mass with left and right lobe hepatectomy, resection of the antrum with Roux-en-Y hepaticojejunostomy, and resection of additional implants in distal ileum, sigmoid, uterus, left ovary, and right perinephric space. Surgical pathology was consistent with stage IV WT (**Images 1**, **2**). Unfortunately, restaging scans 2 months after surgery demonstrated progression of metastases in the spleen, peritoneum, lungs, and liver, causing compression of the inferior vena cava. She had significant elevations in lactate dehydrogenase (LDH) to 437 U/L and alkaline phosphatase to 619 U/L. NGS (Tempus xT platform) from the resected tumor revealed *BRAF V600E* mutation (VAF 45.6%), which was *PDL1* < 1%, and microsatellite instability stable. After discussion at our Molecular Tumor Board, the decision was made to start *BRAF/MEK*-targeted therapy with dabrafenib and trametinib. A repeat scan 3 months after starting *BRAF/MEK* therapy demonstrated regression of her metastatic disease in the liver, spleen, and peritoneum, and decompression of her inferior vena cava with marked clinical and radiographic improvement (**Figure 1**). In the 5 months following initiation of therapy, the patient had a 9.5 kg weight gain, improvement in her abdominal pain, and subjective improvement in her functional status and quality of life. Her previously elevated LDH and alkaline phosphatase at 437 U/L and 619 U/L normalized to 192 U/L and 109 U/L, respectively.

**Figure 2:**
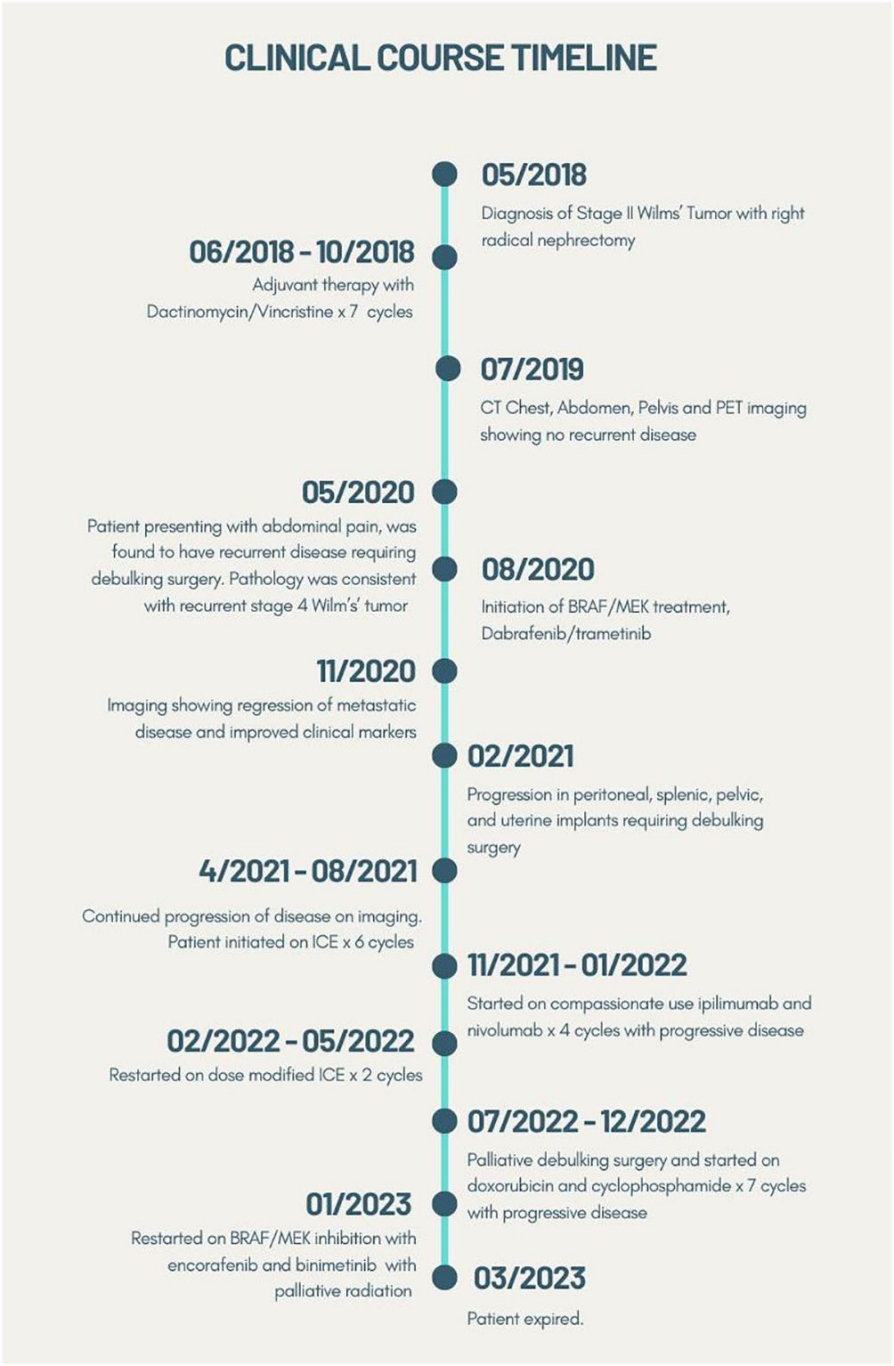
Clinical course of patient.

Although she was feeling improved, restaging scans obtained 5 months into *BRAF/MEK* therapy showed a progression in peritoneal, splenic, pelvic, and uterine implants but a decrease in metastatic disease in the liver and psoas. Due to the uterine implants and extensive tumor burden, she underwent further debulking surgery with bowel resection and anastomosis, hysterectomy, and bilateral salpingo-oophorectomy. Repeat genomic profiling of the blood (Tempus xF platform) demonstrated a retained *BRAF V600E* mutation, as well as a new *KRAS G13D* mutation. The patient was on dabrafenib and trametinib 8 months until, unfortunately, restaging scans revealed new and enlarged lesions in the liver, spleen, and peritoneum suggestive of progressive disease. This was followed by six cycles of ICE (ifosfamide, carboplatin, etoposide) with good partial response, however, experienced complications of myelosuppression and then progressive disease. She was initiated on four cycles of compassionate use ipilimumab and nivolumab and had progressive disease, and then later restarted on dose-modified ICE for two cycles but could not tolerate the therapy. She then received doxorubicin and cyclophosphamide for seven cycles and had partial response, but then progressive disease. Given lack of other options she was restarted on *BRAF/MEK* inhibition with encorafenib and binimetinib along with palliative radiation to pelvic recurrence. She continued to have progression of her hepatic and peritoneal metastases on encorafenib/binimetinib and began discussions to begin therapy with carboplatin and etoposide. Unfortunately, the patient was hospitalized with septic shock and transitioned to hospice care and ultimately expired. This clinical course is summarized in timeline form in **Figure 2**.

## Methods

This study was approved by our institution’s institutional review board at Rush University Medical Center. The patient provided informed consent for this publication.

## Discussion

Although WT is relatively common in the pediatric population, diagnoses in adults are relatively rare without clear guidelines for treatment. A standardized approach to adults has been proposed by the International Society of Pediatric Oncology and Children’s Oncology Group, which is adopted from pediatric guidelines, has been proposed. This protocol includes a multimodal approach to treatment, but outcomes following this guideline remain limited due to the rarity of cases (6). In many cases, adult WT treatment is driven by histological classification, molecular analysis, and staging.

Some of the most common genetic alterations of pediatric Wilms tumor include *IGF2* overexpression, *WT1*, and *CTNNB1* mutations. It is unclear if the genomic landscape of adult tumors differs from the pediatric disease due to a limited number of cases, but *BRAF* mutations remain rare in the pediatric population (7). However, there is one cohort study of 14 adult WT patients with similar common mutations to pediatric WT such as *WT1*, suggesting that they may be driven by similar molecular pathways (8). In this same study, 36% of the patients had *BRAF V600E* mutations, suggesting increased prevalence among adult patients compared to pediatric patients (8).

Metanephric adenoma is thought to be a differentiated form of epithelial predominant WT and have histologic similarities. *BRAF V600E* mutations are present in 90% of metanephric tumors (9). However, *BRAF* mutations in WT are relatively rare with only small cohort studies and cases describing this mutation in WT, some of which were associated with WT/MA overlap tumors (3, 8, 10). Identification of this mutation holds therapeutic significance considering the emergence of targeted therapies against *BRAF*-mutated cancers.

We describe the first case of dual *BRAF/MEK* inhibition in adult WT, which the patient had a clear clinical benefit with improvement in her weight and pain and initial radiologic response to the dual inhibition therapy with evident initial regression of metastasis in liver, peritoneum, and spleen. However, this robust response was only transient has she had progression in her peritoneal, splenic, pelvic, and uterine implants 5 months later in therapy. This response is consistent with the two previously reported cases, which suggests dual *BRAF/MEK* therapy should be considered in patients with *BRAF*-mutated WT.

NGS has expanded treatment options for patients with various malignancies by identifying actionable gene mutations, such as *BRAF/MEK*, where targeted therapy can be utilized, particularly for rare tumors, which may have limited standard-of-care treatment options. Activating mutations in *BRAF*, most commonly the *BRAF V600E* mutation, lead to increased cell survival through increased *BRAF* kinase activity, *MAPK* activity, *MEK* activity, and subsequent *ERK* signal activation (11, 12). Inhibition of the *BRAF* pathway has proven to be an effective target in multiple tumor types expressing this mutation. In a histology-independent trial of the *BRAF* inhibitor vemurafenib, a cohort of patients with non-squamous cell lung carcinoma harboring the *BRAF V600E* mutation demonstrated a 42% partial response rate after treatment with vemurafenib (13). The NCI-MATCH trial has also showed robust response in *BRAF* inhibition with dabrafenib/trametinib. Cohorts of the BRF117019 (ROAR trial) showed response in 33% of high-grade and 69% of low-grade glioma patients, EAY131-H (Subprotocol H) showed an ORR 38% compared with a null rate of 5% (*p* < 0.0001) in patients with various solid tumors, and pediatric CTMT212x2101 trials showed ORR in 76.9% and 58.3% in monotherapy and combination studies, respectively (14–16). Given these robust responses, the Food and Drug Administration (FDA)–approved dabrafenib/trametinib in June of 2022 for tumor-agnostic use in *BRAF*-mutated solid tumors.

*BRAF* inhibitor monotherapy often fails due to acquired resistance and paradoxical *MEK* activation (17, 18). To prevent this resistance, combination therapy of *BRAF/MEK* inhibition has been utilized in many cancers harboring *BRAF V600E* mutations. In a stage III trial of dabrafenib-trametinib versus monotherapy with dabrafenib in mutated *BRAF V600E/*K melanoma, dual therapy was shown to have improved progression-free survival of 9.3 versus 8.8 months, response rate of 67% versus 51%, and overall survival at 6 months of 93% versus 85% when compared to dabrafenib monotherapy (19). The promising results in *BRAF* mutant melanomas have led to trials of dual dabrafenib plus trametinib therapy in non-small cell lung cancer. Studies limited to stage II clinical trials have shown dual therapy has a durable response of 18.2 months versus 12.7 months overall survival of monotherapy. However, dual therapy has also been shown to have significantly increased adverse effects when compared to monotherapy, which led to early discontinuation in 12% of the patients in the dual therapy group compared to 6% in the monotherapy group (20).

Numerous resistance mechanisms to *BRAF/MEK* inhibition have been described—many involving upregulating or activating mutations in the *RAS/RAF/MEK/ERK* pathway or the activation of cell growth pathways involved in cross-talk such as *PI3K/AKT/mTOR* (21). In the described case, we postulate that the acquired *KRAS G13D* mutation following progression on dabrafenib/trametinib represents a resistance mechanism for *BRAF/MEK* inhibition. This mechanism has been described in two published case reports of patients treated with *BRAF/MEK* inhibitor therapy: one patient with papillary thyroid cancer and one patient with non-small cell lung cancer. Interestingly, patients in both case reports developed a *G12V* mutation, but our patient developed a *G13D* mutation (22, 23). Since *KRAS* is upstream of both *BRAF* and *PI3K* pathways, the acquired *KRAS* mutation shunts cellular signaling through the *PI3K* pathway resulting in cell proliferation. Incorporation of *KRAS* inhibitors in this setting makes biological sense but would likely be limited by both toxicity and therapeutic spectrum with the only FDA-approved agent, sotorasib, targeting the *G12C* mutation.

Multiple case reports of successful treatment with vemurafenib monotherapy in adults with WT harboring *BRAF V600E* mutations and case studies demonstrating the efficacy of *BRAF/MEK* dual inhibition in pediatric WT have been published (4). We report the first case of *BRAF V600E* targeted dual therapy using *BRAF/MEK* inhibition with dabrafenib/trametinib in an adult with metastatic recurrent epithelial WT. The remarkable regression in metastases on imaging demonstrates the importance of obtaining information of the molecular landscape of the tumor to identify personalized treatments for patients with more rare tumors.

*BRAF*-targeted therapy is an effective treatment modality for adult WT harboring *BRAF V600E* mutation (3, 11). Given the lack of treatment options available, we encourage continued research for targetable mutations thereby prolonging survival in rare tumors, such as adult WT patients. It is also imperative to continue research on exploring other therapeutic options as majority of patients of *BRAF/MEK* inhibitors tend to relapse within 1 year due to resistance mechanism usually involving *MAPK* pathway activation, which are still poorly understood (24, 25). Leveraging concepts similar to the NCI-MATCH and ComboMATCH, patients with rare cancers such as adult WT should consider tumor genetic profiling to identify actionable alterations with findings shared among other clinicians to help identify which mutations are associated with higher risk and assist in creating appropriate treatment protocols accordingly. Another possible avenue of research is immune checkpoint inhibitors. Unfortunately, we are yet to see a similar response in pediatric solid tumors, including WT, due to very low *PDL-1* expression in pediatric population (26, 27). However, the potential role of *BRAF* inhibitors in modulating the immune microenvironment leading to enhanced efficacy of immunotherapy when combined with *BRAF* inhibitors is gaining interest (11, 24).

## Conclusion

We present the first case to our knowledge of a recurrent adult WT being treated with dual-*BRAF/MEK* targeted therapy, which showed good clinical response and was well tolerated. This patient had an initial robust regression in extensive metastatic disease evidenced by clinical and radiographic improvement in disease markers and tumor burden. She was able to continue *BRAF* inhibitor therapy for over 1 year with improvement in quality of life and progression-free survival before ultimately progressing. Although this patient did ultimately progress, her clear initial response to treatment underscores the importance of targeted therapies in rare tumors. Further studies are needed to navigate other treatment options including targeted therapy and possible synergistic combination regimens, including mechanisms of resistance. *BRAF/MEK* inhibitors should be considered as a treatment option in patients with metastatic WT and *BRAF V600E/K* mutation.

## Data availability statement

The original contributions presented in the study are included in the article/**Supplementary Material**. Further inquiries can be directed to the corresponding author.

## Ethics statement

Written informed consent was obtained from the individual(s) for the publication of any potentially identifiable images or data included in this article.

## Author contributions

MK: Writing – review & editing, Writing – original draft, Validation, Investigation, Formal analysis. CA: Writing – review & editing, Writing – original draft, Validation, Investigation, Formal analysis. JS: Writing – review & editing, Supervision, Investigation, Conceptualization. TC: Writing – review & editing. SP: Writing – review & editing. AT: Writing – review & editing, Validation, Supervision, Investigation, Conceptualization.

## Supplementary material

Image 1 Intra-operative photo of Wilms’ tumor, measuring 26.5 cm × 25.0 cm × 7.2 cm.

Image 2 Histology of removed tumor showing biphasic proliferation of undifferentiated blastemal cells with hyperchromatic nuclei, consistent with Wilms’ tumor.
