# Supplementary figures and images for: Case report: Metastatic BRAF V600E–mutated adult Wilms’ tumor with robust response to BRAF/MEK inhibitor therapy
Source: Front Oncol. 2024 Jul 15;14:1376270. doi: 10.3389/fonc.2024.1376270 (PMC11373342; doi:10.3389/fonc.2024.1376270)

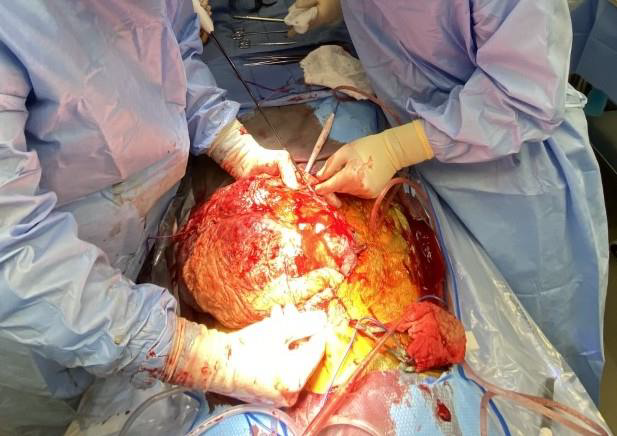

Supplement: Image 1 — Intra-operative photo of Wilms’ tumor, measuring 26.5 cm × 25.0 cm × 7.2 cm. [file Image_1.tiff]

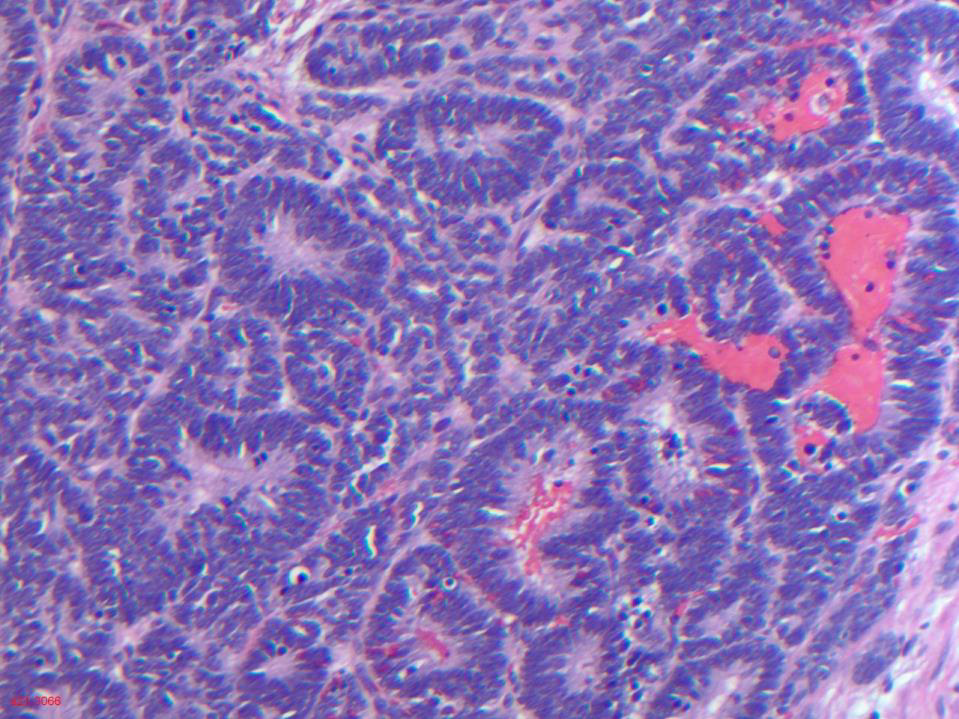

Supplement: Image 2 — Histology of removed tumor showing biphasic proliferation of undifferentiated blastemal cells with hyperchromatic nuclei, consistent with Wilms’ tumor. [file Image_2.tiff]
